# Long‐distance migrants vary migratory behaviour as much as short‐distance migrants: An individual‐level comparison from a seabird species with diverse migration strategies

**DOI:** 10.1111/1365-2656.13431

**Published:** 2021-02-09

**Authors:** J. Morgan Brown, E. Emiel van Loon, Willem Bouten, Kees C. J. Camphuysen, Luc Lens, Wendt Müller, Chris B. Thaxter, Judy Shamoun‐Baranes

**Affiliations:** ^1^ Institute for Biodiversity and Ecosystem Dynamics University of Amsterdam Amsterdam The Netherlands; ^2^ Department of Coastal Systems NIOZ Royal Institute for Sea Research and Utrecht University Texel the Netherlands; ^3^ Terrestrial Ecology Unit Ghent University Ghent Belgium; ^4^ Behavioural Ecology and Ecophysiology Research Group University of Antwerp Antwerp Belgium; ^5^ British Trust for Ornithology Norfolk UK

**Keywords:** GPS tracking, individual differences, migration, movement ecology, phenology, plasticity, repeatability, seabird

## Abstract

1. As environmental conditions fluctuate across years, seasonal migrants must determine where and when to move without comprehensive knowledge of conditions beyond their current location. Animals can address this challenge by following cues in their local environment to vary behaviour in response to current conditions, or by moving based on learned or inherited experience of past conditions resulting in fixed behaviour across years.
2. It is often claimed that long‐distance migrants are more fixed in their migratory behaviour because as distance between breeding and wintering areas increases, reliability of cues to predict distant and future conditions decreases. While supported by some population‐level studies, the influence of migration distance on behavioural variation is seldom examined on an individual level.
3. Lesser black‐backed gulls *Larus fuscus* are generalist seabirds that use a diversity of migration strategies. Using high‐resolution multi‐year GPS tracking data from 82 individuals from eight colonies in Western Europe, we quantified inter‐ and intra‐individual variation in non‐breeding distributions, winter site fidelity, migration routes and timing of migration, with the objectives of determining how much variation lesser black‐backed gulls have in their migratory behaviour and examining whether variation changes with migration distance.
4. We found that intra‐individual variation was significantly lower than variation between individuals for non‐breeding distributions, winter site fidelity, migration routes and timing of migration, resulting in consistent individual strategies for all behaviours examined. Yet, intra‐individual variation ranged widely among individuals (e.g. winter site overlap: 0–0.91 out of 1; migration timing: 0–192 days), and importantly, individual differences in variation were not related to migration distance.
5. The apparent preference for maintaining a consistent strategy, present in even the shortest distance migrants, suggests that familiarity may be more advantageous than exactly tracking current environmental conditions. Yet, variation in behaviour across years was observed in many individuals and could be substantial. This suggests that individuals, irrespective of migration distance, have the capacity to adjust to current conditions within the broad confines of their individual strategies, and occasionally, even change their strategy.

## Graphical abstract

This study uses multi‐year high resolution GPS tracks of lesser black‐backed gulls to quantify variation in migration behaviour at the individual‐level and along broad range migration distances to demonstrate that long‐distance migrants are no more fixed in their migratory behaviours than short distance migrants, contrasting with findings from population‐level studies.

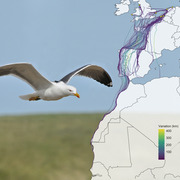

## 1 INTRODUCTION

Seasonal environments offer animals predictable periods of high productivity, though also presenting the challenge of scarcity during the other portion of the annual cycle. Seasonal migration is a life‐history strategy by which animals can exploit fluctuations in habitat suitability by moving between distant regions at predictable times throughout the year (Shaw & Couzin, 2013). Animals that match their movements more precisely to coincide with environmental patterns in their landscape (e.g. food, weather) typically have higher survival and reproductive success (Both et al., 2006). Environmental conditions, however, vary unpredictably among years. This poses a challenge for migrants who must determine when and where to move without comprehensive knowledge of the environment through which they must move.

When environmental conditions are spatially and temporally autocorrelated (Koenig, 1999), conditions at one location can provide information about conditions in another. Such correlations can be used as cues for migratory behaviour (Saino & Ambrosini, 2008), but as the distance an animal migrates increases, the correlation of conditions between wintering and breeding areas, and thus the reliability of these cues, is expected to decrease. A lack of reliable information favours tracking of past conditions, that is, average long‐term trends), rather than responding to current conditions (Bauer et al., 2020). Due to these differences in the availability and reliability of cues for predicting remote conditions, it is commonly suggested that long‐distance migrants should be more fixed in their migratory behaviour than short‐distance migrants (Gwinner, 1977; Hagan et al., 1991, reviewed by Knudsen et al., 2011).

Long‐distance migrants are thus expected to move based on learned (Campioni et al., 2020), socially transmitted (Jesmer et al., 2018) or genetically inherited (i.e. endogenous, Åkesson et al., 2017; Berthold, 1996; Gwinner, 2003) information about spatiotemporal resource availability in the past. Movement based on past information is synonymous to memory‐based movement (Fagan et al., 2013), and movements should coincide with average climatic conditions (Abrahms et al., 2019; Thorup et al., 2017). Using past information should result in low intra‐individual variation in time and space across years, and consistent differences in behaviour between individuals. Short‐distance migrants, on the other hand, are generally expected to adjust migratory behaviour based on current conditions, resulting in intra‐individual variation across years. This may be done by either following current resource gradients (i.e. surfing resource waves, Armstrong et al., 2016; Van der Graaf et al., 2006) or using local environmental cues such as temperature (Deutsch et al., 2003) or vegetation (Balbontín et al., 2009; Merkle et al., 2016; Van der Graaf et al., 2006) to predict remote and future resource patterns. Explicit laboratory experiments for differential information use by migration distances have not been performed, while support from inter‐species comparisons of variation in phenology of wild populations is mixed (Knudsen et al., 2011): most report that timing of migration in long‐distance migrating species is less varied than short‐distance ones (Butler, 2003; Hagan et al., 1991; La Sorte et al., 2016; Miller‐Rushing et al., 2008; Murphy‐Klassen et al., 2005; Rainio et al., 2006; Rubolini et al., 2010), while others observe no differentiation or even more advancement in long‐distance migrants (Hüppop & Hüppop, 2003; Jonzén et al., 2006).

Most field‐based studies examining the influence of migration distance on variation in migration behaviour occur at the population level (Charmantier & Gienapp, 2014), and the extent to which individual‐level behavioural plasticity contributes to population‐level changes in migratory behaviour remains unclear (Knudsen et al., 2011). Repeated‐measures of migratory traits from individuals to measure inter‐ and intra‐individual variance is a commonly used method to assess plasticity in migratory behaviour (Conklin et al., 2013; Fraser et al., 2019). Consistent individual differences, or repeatability, may be indicative of inherited or learned preferences based on past conditions, while the residual within‐individual behavioural variability reflect the combination of plastic responses to the environment (i.e. adjustment to current conditions) and flexibility (i.e. variation independent of the environment; Hertel et al., 2020; Nakagawa & Schielzeth, 2010; Noordwijk et al., 2006). While repeatability of migratory behaviour has been calculated previously for many avian species (reviewed by Both et al., 2016; Phillips et al., 2017), typically the spatial accuracy of these studies are low due to the tracking technology used, and comparisons among individuals or populations using different strategies are seldom carried out. It is therefore challenging to compare results across these studies to understand the ultimate ecological cause for differences in behavioural variation across taxa (Charmantier & Gienapp, 2014). Species containing individuals with different migratory strategies are interesting systems for examining whether migration distance influences individual variation in migratory behaviour.

Lesser black‐backed gulls *Larus fuscus* are medium‐sized, long‐lived seabirds that migrate to diverse wintering regions. A single colony typically contains individuals ranging from short‐distance migrants that remain local and only move to winter roosting sites 50 km away, up to intercontinental long‐distance migrants travelling thousands of kilometres (Shamoun‐Baranes et al., 2017; Stienen et al., 2016; Thaxter et al., 2019). An individual's wintering region is thought to be consistent across years and is not related to either sex or size (Baert et al., 2018). Lesser black‐backed gulls are capable of using a range of resource types, including marine, terrestrial and urban (Baert et al., 2018; Camphuysen et al., 2015), though within a given period, many individuals tend to specialize on a particular foraging strategy (Camphuysen et al., 2015; Isaksson et al., 2016). Having the capacity to forage in a broad range of habitats and survive in a range of climatic conditions provides many potential options with regards to how, when and where they migrate.

Using a long‐term, high resolution GPS‐tracking dataset of lesser black‐backed gulls breeding in colonies in Belgium, the UK and the Netherlands, with individuals that have been tracked for multiple years, we measured variation in the following migratory behaviours: non‐breeding distribution, fine‐scale wintering site fidelity, migratory routes and date of arrival and departure from breeding and wintering areas. One of the advantages of our study system is the high spatio‐temporal resolution of our data across all colonies (hourly at ±3 m spatial resolution) which enables us to accurately quantify at a fine spatio‐temporal scale each of the migratory behaviours we studied. Our first objective is to quantify inter‐ and intra‐individual variation of these migratory behaviours in lesser black‐backed gulls and determine whether individuals use consistent strategies. While a range of behavioural options may be available to an individual, there are benefits to behaving consistently in space and time (Gunnarsson et al., 2004; Piper, 2011). Thus, we hypothesize that individuals will generally be consistent in their migratory behaviour, with population variation being largely a result of inter‐individual differences. Our second objective is to determine whether individual variation in migratory behaviour changes with migration distance. Studying variation in migration behaviour at the individual‐level, a high spatio‐temporal resolution and along such a broad range migration distances has rarely been possible, allowing us to address this question from a new ecological perspective.

## 2 MATERIALS AND METHODS

### 2.1 Tracking and data processing

**FIGURE 1:**
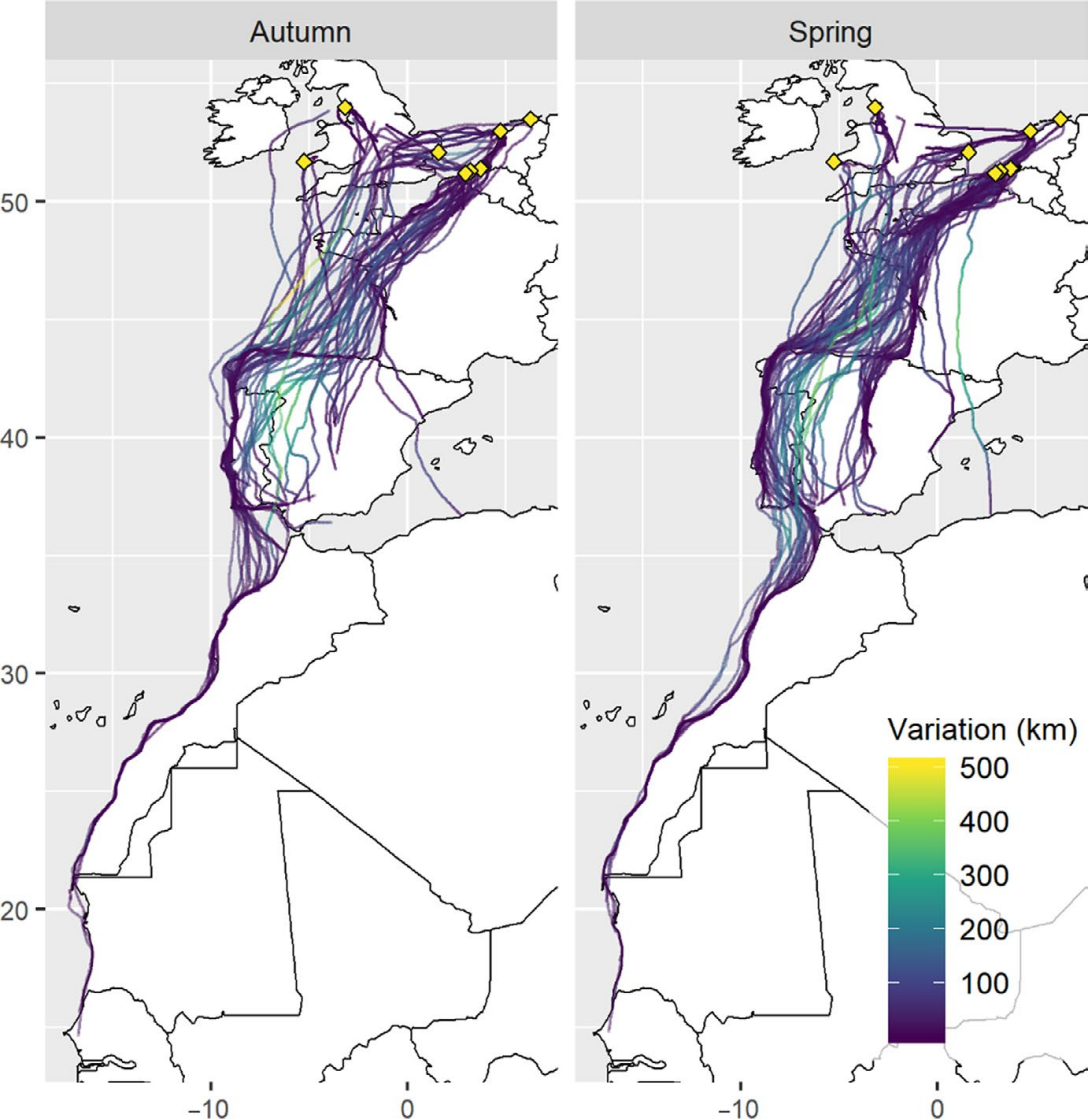
Mean individual migration routes in autumn and spring based on GPS tracking from two or more years. Variation around the mean route is shown by colour. Colonies are indicated with yellow diamonds

We used GPS tracking data from adult lesser black‐backed gulls tracked for two or more years from eight colonies in the Netherlands, Belgium and the UK (Table 1, Figure 1). Gulls were captured during the breeding season using walk‐in traps set over the nest during incubation. Subsequent movements were recorded using solar‐powered GPS‐trackers (UvA‐BiTS, Bouten et al., 2013), attached with a Teflon wing harness (Thaxter et al., 2014). Total mass of tracker and harness were less than 3% of total body mass.

**TABLE 1:** Summary of data included in this study by colony. Number of individuals (N. ind.) and number of seasons (N. seasons) used in the analysis are reported as: ‘overlap and timing’/‘autumn routes’/‘spring routes’

Colony	Location	Years	N. ind.	N. seasons
Walney, UK	54.0 N, 3.18 W	2014–2019	11/6/6	28/14/15
Orfordness, UK	52.1 N, 1.58 E	2010–2015	9/1/7	22/3/18
Skokholm, UK	51.7 N, 5.27 W	2014–2017	5/4/4	11/9/9
Schiermonnikoog, NL	53.5 N, 6.26 E	2017–2019	4/3/4	8/6/8
Texel, NL	53.0 N, 4.72 E	2010–2019	18/13/15	61/33/44
Vlissingen Oost, NL	51.4 N, 3.70 E	2015–2019	9/8/9	24/22/24
Zeebrugge, B	51.3 N, 3.18 E	2013–2019	24/17/22	74/54/67
Oostende, B	51.2 N, 2.93 E	2016–2019	2/2/2	5/5/5
Total			82/54/69	233/146/190

The breeding season was defined as the period of the year during which an individual occurs in the breeding colony, regardless of their breeding status. The non‐breeding season therefore starts with date of colony departure (last detection within 10 km of the breeding colony following the breeding season) and continues until date of colony arrival (first detection within 10 km of the colony prior to the breeding season). To quantify time spent in different areas throughout the non‐breeding season (non‐breeding distribution), we calculated a utilization distribution (UD) from the 95% kernel density estimates of GPS locations taken during the non‐breeding season. Tracking data were subsampled to a 12‐hr interval to reduce autocorrelation and help distribute data equally through time (in the case of multi‐day data gaps) and were projected onto a Lambert equal‐area projection (EPSG 3035). UDs were created using the r package ‘adehabitatHR’ (Calenge, 2006) with a bivariate normal kernel on a grid with a 10‐km resolution, using a fixed bandwidth (*h*) of 100 km.

Gulls can use several distinct core areas over the course of a non‐breeding season. These **core areas** were identified by polygons of the 50% contour from the non‐breeding distribution UD (see Figure  S1 for examples). Core areas identify coarse‐scale regions (hundreds of kilometres in diameter) where birds either wintered or stopped‐over for prolonged periods.

Many non‐breeding seasons contained multi‐day gaps caused by low battery or device malfunction which can influence the UD. Any non‐breeding season with a consecutive gap longer than 21 days (the minimum time spent in a core area from gap‐free seasons) was removed. If these removals resulted in an individual with only one remaining season, this individual was removed from the study. One individual who remained within 10 km of its colony year‐round was also removed.

The core area in which an individual spent the most amount of time between December and March was considered the wintering area, and apart from one individual, was the furthest core area from the colony. Date of arrival to wintering area and date of departure from wintering area were the dates of the first and last GPS detection within this polygon, respectively. The remaining core areas are considered to represent stopover areas. Time spent in these stopover areas sometimes exceeds time spent in the wintering area, and these areas are typically occupied in summer and autumn months (Klaassen et al., 2012). Migration distance, representing the migration strategy of an individual (i.e. direct rather than cumulative distance travelled), was measured as the great circle distance between the colony and centroid of the wintering area. For four individuals, the wintering area from 1 year was overlapping with multiple small polygons in another year. To make behaviour comparable across years, these fragmented polygons were grouped into single wintering areas.

### 2.2 Non‐breeding distribution

To quantify intra‐individual variation in non‐breeding distributions, we calculated mean overlap in the 95% non‐breeding season UDs (described above) between all possible paired combinations of non‐breeding seasons per individual using Bhattacharyya's affinity (BA, Bhattacharyya, 1943), a recommended method for quantifying home‐range overlap (Fieberg & Kochanny, 2005). BA is a function of the product of two UDs which quantifies their similarity, with 0 indicating no overlap and 1 being identical (as we are using 95% UDs, 0.95 would be the highest potential overlap). This metric is independent of area so is comparable across areas of different size (i.e. consistent use of a concentrated area ranks the same as consistent use of a larger, diffuse area). Because BA uses the complete probability distribution, individuals overlapping in areas with higher probability of occurrence (i.e. similar use of wintering and stopover sites across years), will have higher overlap than those overlapping in areas of low probability (i.e. if stopping over in different areas or for less time).

Inter‐individual variation in non‐breeding distribution was quantified by calculating non‐breeding season overlap between pairs of individuals using similar migration strategies. Pairings were constrained so that neither the breeding colonies nor wintering areas used by paired individuals were further than 250 km apart. The 250 km constraint was chosen a‐priori to any statistical analysis, and was selected because, considering the motion capacity of this species, the area within a 250‐km range represent accessible alternatives for an individual while being large enough that most individuals could be paired to at least one other individual. The paired tracks were not required to be from the same year, and if multiple nonbreeding seasons were within the distance constraints for a pair of individuals, one non‐breeding season per individual was randomly selected.

Following Guilford et al. (2011), to determine if individuals were significantly more consistent in their behaviour across years relative to the behaviour demonstrated by others, we used randomization tests. First, the difference between median variation between pairs of individuals and median variation within individuals was calculated. The data were then randomly re‐arranged into new ‘between’ and ‘within’ groups and the difference between medians of these random groupings was found. Randomizations were repeated 10,000 times. The probability of the difference in medians from randomly generated groups being larger than that found between the actual within‐individual and between‐individual groups was then reported.

The relationship between migration distance and intra‐individual variation in non‐breeding season distribution was examined using a linear model of non‐breeding season overlap against the median migration distance used by each individual. Individuals who had a wintering area which did not overlap with previous years were excluded from this, and all other comparisons of the influence of migration distance on intra‐individual variation, so that the measured intra‐individual variation could be associated with a single migration distance and wintering area. A likelihood ratio test between this model and a model with no explanatory variables was used to test whether migration distance significantly influenced individual variation.

However, short‐distance migrants are more constrained in how much they can reasonably change their behaviour, and thus should demonstrate less variation regardless of their inclination for behavioural variation. To address this bias, the relationship between migration distance and overlap found between paired‐individuals was used as a null model for expected variation at a given migration distance, assuming inter‐individual variation should be similarly influenced by this spatial constraint. The variation predicted in this null model for a given migration distance was subtracted from the intra‐individual non‐breeding season overlap to determine whether intra‐individual variation changed more or less than expected.

### 2.3 Winter site fidelity

As a measure of consistency in fine‐scale space use, we calculated winter site fidelity. All GPS points between arrival and departure from the wintering area were used to maximize temporal resolution of movement data, rather than subsampling as done for non‐breeding distributions. The biased random bridge approach was used to calculate a **winter area UD**, which considers the sampling interval of GPS points thus accounting for spatio‐temporal autocorrelation in high frequency measurement schemes (Benhamou, 2011). Winter area UDs were calculated on 500‐m^2^ grids using the BRB function in the r package ‘adehabitatHR’, with the plug‐in method for estimating the diffusion coefficient. The maximum duration was set to 3 hr, with a minimum distance of 20 m and a minimum smoothing parameter of 150 m. Site fidelity was then calculated using BA overlap of the winter area UD up to the 95th percentile, which is used as a measure of individual consistency (Abrahms et al., 2018; Wakefield et al., 2015).

A linear model of winter site fidelity against individual median migration distance was fit and compared to a model with no fixed effects to test whether migration distance significantly influences winter site fidelity (excluding individuals who changed wintering areas).

### 2.4 Migratory routes

To quantify variation in migration routes, defined as the path recreated from the GPS track of an individual migrating between wintering and breeding areas, we computed an autumn and spring mean route for each individual. Based on a method by Freeman et al. (2010), the mean route is a sequence of 500 computed points that minimizes the distance to nearest‐neighbour locations on the set of GPS tracks used by an individual across years. All points within stopover areas were removed and replaced by a single point at the centroid of the stopover area polygon so that these routes are only composed of GPS points from migratory flights. The variance of nearest‐neighbour locations from the GPS‐tracked migration routes around each point on the mean route was calculated, and the mean of the variances along the mean route was used as a metric of **migration route variation**. Any migration route with a gap longer than 24 hr when an individual was outside of a core area (i.e. during migratory flights) was removed, as were individuals who changed wintering area. Full methodology and illustrated example of mean route and migration route variation calculations are in the Supporting Information (‘*Methodology: Calculation of mean routes’,* Figure S2).

Inter‐individual variation in migration routes was calculated using the same between‐individual pairing method used for non‐breeding season overlap, and individual consistency was determined using randomization tests, as described above. Influence of migration distance on migration route variability was assessed using linear models for each season. Similar to non‐breeding season overlap, short‐distance migrants are expected to be more spatially constrained than long‐distance migrants, so this relationship was also considered in comparison to that found for between‐individual pairings.

### 2.5 Timing of migration

As measures of intra‐individual variation in annual timing we report the range of dates individuals departed and arrived at their colony and wintering areas. One individual was removed from analysis of departure from colony and two individuals from arrival to wintering area as data gaps occurred during this transition.

To quantify individual consistency in timing we calculated repeatability, *R*, where

$$R=\frac{s_{a}^{2}}{s_{a}^{2}+s_{\varepsilon }^{2}},$$

and $s_{a}^{2}$ and $s_{\varepsilon }^{2}$ are the variance among and within individuals, respectively. If individuals are highly consistent in their behaviour relative to variation occurring among individuals, *R* is close to one. We calculated $s_{a}^{2}$ and $s_{\varepsilon }^{2}$ using linear mixed models (LMM) for each trait, with migration distance as a fixed effect and colony and individual as random effects (REML method using lme4 package in r, Bates et al., 2015), where variance of the individual‐level random effect is $s_{a}^{2}$ and variance of the random error is $s_{\varepsilon }^{2}$ (Nakagawa & Schielzeth, 2010). For arrival to wintering area, migration distance was excluded to achieve model convergence. As we were interested in the degree of behavioural variation an individual could exhibit, year was not included as a random effect so that behavioural variation in response to inter‐annual changes in environmental conditions would contribute to intra‐individual (residual) variation. We used the r package ‘rptR’ (Stoffel et al., 2017) to calculate repeatability with 95% confidence intervals based on parametric bootstrapping over 1,000 iterations (presented as *R*[Lower CI − Upper CI]).

For individual‐level measures of variation in arrival and departure dates, using the LMMs above, we calculated an individual‐level repeatability, *R_i_*, by substituting the residual variance for the *i*th individual, $s_{i}^{2}$, for $s_{\varepsilon }^{2}$ (excluding individuals who changed wintering areas; Potier et al., 2015; Wakefield et al., 2015). *R_i_* for each arrival or departure was then used as the response variable in the linear models with migration distance.

All analysis was completed in R version 3.5.1. Final sample sizes for each behaviour can be found in Table 1 and Table S1.

## 3 RESULTS

### 3.1 Variation in migratory behaviour of lesser black‐backed gulls

Individuals used between 1 and 3 core areas during the non‐breeding season. For all but five individuals (*n* = 77, 94%), winter areas overlapped across all years. These five individuals switched wintering areas between France and Western Sahara (*n* = 1), Mauritania and Portugal (*n* = 1), France and UK (*n* = 1) and Morocco and UK (*n* = 2). Migration distance was therefore highly repeatable (*R* = 0.81 [95% confidence interval: 0.57–0.93]). Sixty‐two individuals (76%) used a stopover in at least 1 year, and 96% of total time spent in stopover areas occurred before arrival to the wintering area. Use of stopover areas was less consistent than wintering areas: 18 out of the 62 individuals using a stopover (29%) had a stopover area that did not overlap among years (compared to 6% individuals who had non‐overlapping wintering areas).

**FIGURE 2:**
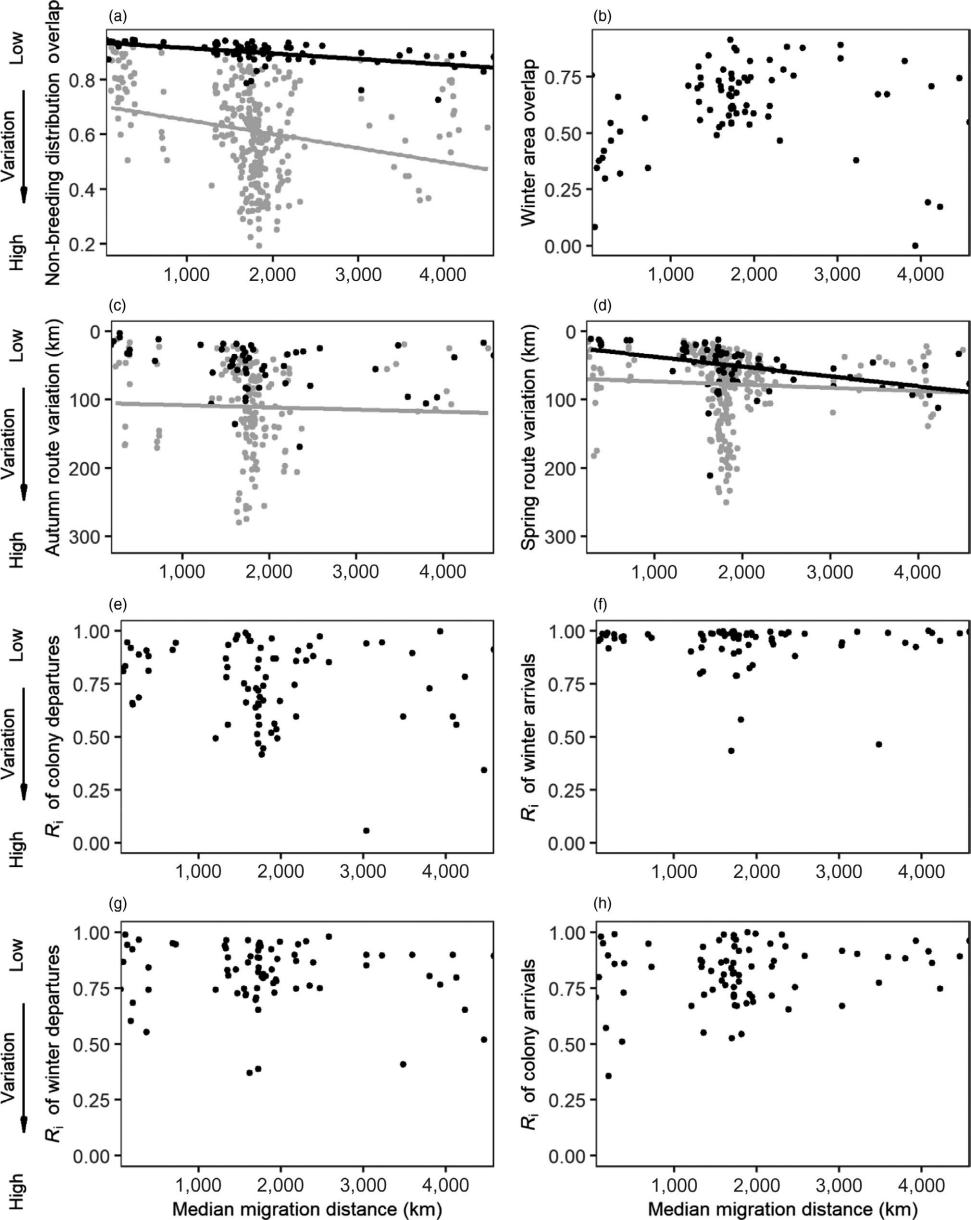
Mean overlap in (a) non‐breeding distribution and (b) winter areas, (c) autumn and (d) spring variation in migration routes, and individual repeatability in date of (e) colony departure, (f) arrival to winter area, (g) departure from winter area and (h) colony arrival, across multiple non‐breeding seasons from lesser black‐backed gulls, versus their median migration distance. The *y*‐axis for autumn and spring route variation (c and d) is reversed so that the order of variation is consistent among plots. Black lines showing trends predicted by the linear models were included if significant. Distributions from between‐individual pairs used to calculate ‘residual’ intra‐individual variation are shown in grey (only used for behaviours compared across multiple spatial scales). Individuals who changed wintering areas (*n* = 5) have been excluded

Despite some variation in stopover area use, overlap in non‐breeding distributions was generally high, with a median overlap of 0.91 (range: 0.51–0.95). Non‐breeding distributions were considerably more similar within individuals across years than between individuals (median between‐individual overlap = 0.61, range = 0.19–0.93, Figure 2a and Figure S3), with none of the randomized sets producing a difference in medians more extreme than the actual data (*p* < 0.001). Site‐fidelity within wintering areas was lower than non‐breeding distribution overlap, with a median overlap of 0.62, and differed substantially among individuals (range: 0.00–0.91, Figure 2b).

Individual variation in migration route was generally low in both autumn (median = 41 km, range = 3–169) and spring (median = 45 km, range = 11–211). Most intra‐individual route variation occurred at the Bay of Biscay and over the arid centre of Spain (Figure 1). Intra‐individual variation was significantly lower than variation found between paired individuals (autumn: between‐individual median = 107 km, range = 12–280; spring: between‐individual median = 62 km, range = 11–236; Figure 2c,d and Figure S3) with probabilities <0.001 of obtaining a difference in medians as extreme from a randomized set. Examples of high and low non‐breeding distribution overlap and route variation for a range of migration distances can be found in the Supporting Information (Figure S1 and S4).

**FIGURE 3:**
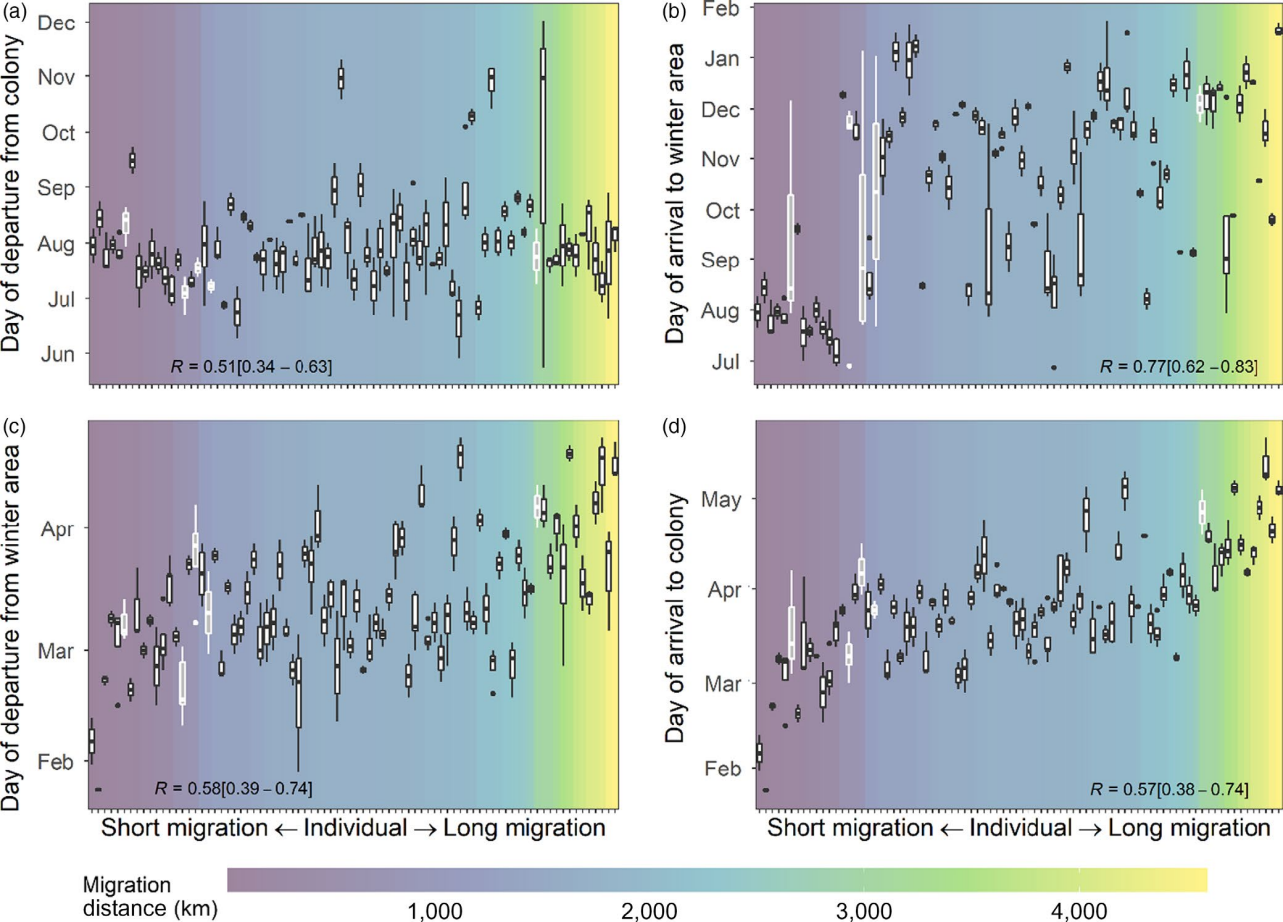
The range of (a) departure dates from colony, (b) arrival dates to wintering area, (c) departure dates from wintering area and (d) arrival dates to colony used across non‐breeding seasons by individual lesser black‐backed gulls. Individuals who changed wintering areas (*n* = 5) are identified by white boxplots. Individuals are ordered by their median migration distance. Repeatability [95% confidence interval] is reported at the bottom of each plot

Individuals departed their breeding colonies within a 192‐day period between 24 May and 2 December (Figure 3a). The median range of departure dates within an individual was 20 days (range: 0–192) and was highly repeatable (*R* = 0.51 [0.34–0.63]). Arrival to wintering area occurred between 27 June and 24 January (a period of 211 days; Figure 3b). Intra‐individual arrival dates to wintering area ranged across years by 1–166 days (median = 16.5) and repeatability was high (*R* = 0.77 [0.62–0.83]). During spring, the range of departure dates from wintering areas and arrival dates to colony within individuals was narrower than in autumn. Departure dates from wintering areas occurred within a 90‐day period between 24 January and 24 April (Figure 3c). The median intra‐individual range of departure dates was 11 days (range: 1–36). Repeatability of departure from wintering area was high (*R* = 0.58 [0.39–0.74]). Arrival to breeding colonies occurred between 24 January and 21 May (a period of 117 days; Figure 3d). Intra‐individual range in arrival dates to colony was 0–34 days (median = 10). Repeatability was also high (*R* = 0.57 [0.38–0.74]). Except for departure date from colony, migration dates were later in longer distance migrants (Figure 3, Table S2). Colony explained little variance in timing of migration. Linear mixed model results and partitioning of variance are reported in the Supporting Information (Table S2).

While most individuals tended to be highly consistent, for each behaviour, a few individuals demonstrated high variation (Figure 2, Figure S5). The individuals demonstrating the most variation were not the same for each behaviour, with 26 different individuals (32%) being in the upper 5th percentile of variation for at least one behaviour (Table S3).

### 3.2 Influence of migration distance on individual variation

Migration distances ranged from 53 to 4,572 km (median = 1727 km, *n* = 77). Intra‐individual non‐breeding distribution overlap decreased with migration distance (overlap = 0.936–2.0 × 10^−5^·migration distance, *F*_1,75_ = 28.142, *p* < 0.001; Figure 2a). However, intra‐individual variation increased at a significantly lower rate than the increase between individuals (residual overlap = 0.232 + 3.1 × 10^−5^·migration distance, *F*_1,75_ = 68.642, *p* < 0.001; Figure S6a), suggesting that longer distance migrants were less variable in their behaviour than expected when considering the total space traversed during their movements (and vice‐versa for shorter distance migrants). Intra‐individual route variation increased slightly but significantly with migration distance in spring (variation = 22.652 + 0.014·migration distance, *F*_1,67_ = 14.237, *p* < 0.001; Figure 2d), even after accounting for increasing between‐individual variation (variation = −46.208 + 0.010·migration distance, *F*_1,67_ = 6.564, *p* = 0.013, Figure S6c). Autumn route variation did not change with migration distance (*F*_1,52_ = 3.941, *p* = 0.052; Figure 2c), nor did it significantly differ from variation observed between individuals (*F*_1,52_ = 1.544, *p* = 0.220, Figure S6b).

Winter site fidelity did not change significantly with migration distance (*F*_1,75_ = 1.269, *p* = 0.263, Figure 2b). Migration distance also did not correlate with individual repeatability in departure date from colony (*F*_1,74_ = 2.258, *p* = 0.137; Figure 2e), arrival to wintering area (*F*_1,73_ = 0.460, *p* = 0.500; Figure 2f), departure from wintering area (*F*_1,75_ = 1.422, *p* = 0.237; Figure 2g) or arrival to colony (*F*_1,75_ = 3.508, *p* = 0.065; Figure 2h), suggesting individual consistency of migration timing did not increase with migration distance.

## 4 DISCUSSION

This study quantified inter‐ and intra‐individual variation in non‐breeding distributions, winter site fidelity, migration routes and timing of migration in lesser black‐backed gulls at the individual‐level, using high spatio‐temporal resolution tracking data, and covering a broad‐range of migration distances, to test the hypothesis that migratory behaviour should become more fixed as migration distance increases. However, we found that migration distance did not explain which individuals were most variable across years, contrasting with many previous inter‐species comparisons of population phenology. Instead, we found that regardless of migration distance, individuals consistently differed from each other in their behaviour, suggesting that individuals predominantly follow learned and/or inherited behavioural strategies.

### 4.1 Variation in migratory behaviour

For all behaviours examined, intra‐individual variation was small compared to that of the population, resulting in distinct individual behavioural strategies, consistent with our hypothesis that gulls will be inclined to rely on past experience. Repeatability was high in comparison to findings across a range of taxa for diverse behavioural traits (Bell et al., 2009), but consistent with studies of avian migration (reviewed by Both et al., 2016; Phillips et al., 2017). This suggests that many avian species preferentially use learned or inherited knowledge of previously reliable wintering and stopover areas, rather than risk searching for the best locations in a given year.

Winter area overlap demonstrated individuals also had high site fidelity at a fine scale (500 m resolution), suggesting repeated use of foraging areas and roosting sites among years. Individual consistency in space use may provide more stable energetic rewards than plastic behaviour (Abrahms et al., 2018), as familiarity with a site can improve foraging efficiency (Piper, 2011; van den Bosch et al., 2019). Efficiency resulting from familiarity may be sufficient to balance the benefits of switching to a new location with better environmental conditions for a given year. Consistent individual differences in timing of migration may be a result of individual differences in foraging type and habitat quality at their respective wintering and stopover areas, resulting in different optimal migration times (Studds & Marra, 2005), and it may also be a mechanism to reunite with mates in the breeding colony (Gunnarsson et al., 2004). Understanding how these individual strategies are determined (genetically inherited, socially transmitted or learned) is important for assessing the adaptive scope of migratory animals to changes in their environment. Current studies on avian species suggest migratory behaviour may be under strong genetic influence in early life, but refined or replaced by learning as an individual gains experience (Campioni et al., 2020; Sergio et al., 2014).

Inter‐individual variation for most behaviours examined was high. High inter‐individual variation might suggest that selective pressure on these behaviours is low for this species (Verhoeven et al., 2019). Low selective pressure on migratory traits may be typical for generalist species, such as gulls, for whom the ability to use a range of behaviours at fine spatio‐temporal scales (e.g. diet and habitat), and the ability to survive under a range of climatic conditions, may buffer the effects of inter‐annual variation, enabling consistency in behaviours at mid‐to‐broad spatio‐temporal scales (e.g. wintering and stopover regions, migratory period). This is conductive with the fact that spatial overlap measured at finer scales (winter site fidelity) was lower than regional‐scale, non‐breeding season overlap.

While most individuals follow a distinct strategy, the intra‐individual variation observed suggests that gulls still adjust behaviour across years, and thus behaviour is not rigidly fixed. Instead, consistent behavioural strategies likely define a broad window in space or time within which an individual can adjust its behaviour based on current conditions, thus allowing for the integration of information based on both past and current conditions (Åkesson & Helm, 2020). Additionally, for each behaviour examined, there were a few individuals with extremely high variation across years (i.e. a change in the behavioural strategy). The individuals which exhibited this high variation were not consistent across all behaviours, suggesting that the ability to change strategy could be common across all individuals. The causes of these drastic changes are unknown, but suggests that individuals can change strategies to adapt to shifting long‐term conditions within their lifetime.

Intriguingly, for migration routes, inter‐ and intra‐individual variation was low, suggesting the entire population is being constrained to the use of certain migratory corridors. Despite reduced inter‐individual variation, intra‐individual variation was still lower, suggesting individuals travelling between similar breeding and wintering areas consistently use different routes. This is in contrast to many migratory bird species who typically demonstrate high variation in migration routes, presumably as they adjust routes among years to current wind conditions (Dias et al., 2013; López‐López et al., 2014; Stanley et al., 2012). This may suggest that there is high selection pressure for moving along coastlines in this species, implying an advantage to foraging or roosting in coastal habitats while migrating. Coastal areas may also represent energy efficient pathways, as the dunes and cliffs typical of these areas can generate orographic lift enabling gulls to switch from flapping flight to energetically cheap soaring flight (Sage et al., 2019).

### 4.2 Influence of migration distance on individual variation

No clear effect of migration distance on individual variation was found in lesser black‐backed gulls from these populations. This is in contrast to numerous phenological studies, covering a range of avian taxa, which have found that species migrating long distances are more fixed in their timing of spring migration compared to short‐to‐mid‐distance migrants, both in response to long‐term climate change (Hagan et al., 1991; Miller‐Rushing et al., 2008; Murphy‐Klassen et al., 2005; Rubolini et al., 2010) and year‐to‐year changes in environmental conditions (La Sorte et al., 2016; Rainio et al., 2006). However, these phenological studies are inter‐specific comparisons focusing either on population means or ‘first individual’ observations, rather than examining individual‐level variation using repeated measures. Similar to our study, Verhoeven et al. (2019) found no influence of winter region on intra‐individual variation in migration timing. High intra‐individual variation has also been reported for some long‐distance migrants (e.g. Fraser et al., 2019), but not all (e.g. Conklin et al., 2013), providing poor support for a general trend for fixed migratory behaviour in long‐distance migrants at the individual‐level. This highlights the importance of integrating individual‐ and population‐level data to better understand the mechanisms and implications of how species react to changing climates (Visser et al., 2010).

While long‐distance migrants may not have reliable cues regarding remote environmental conditions, they may still adjust their migratory behaviour to changes in their intrinsic state or local conditions. Therefore, similar behavioural variability across individuals migrating different distances does not mean all migrants can respond equally well to environmental variation on short or long time‐scales. To draw such conclusions, deviations from an individual's strategy should be correlated with changes in environmental conditions in breeding areas. Indeed, our study is limited by our inability to relate movement to a single preferred resource, as can be done for dietary specialists (Abrahms et al., 2019; Thorup et al., 2017; Van der Graaf et al., 2006). In the future, a better understanding of the underlying motivation and environmental cues gulls use to inform migratory behaviour would help further elucidate the mechanisms underlying migratory decision making in this species.

Given the readily accessible environmental information available to the shortest distance migrants, it is particularly surprising that we still observed individual consistency in space and time. Conditions on wintering areas are typically thought to be less reliable at higher latitudes (Danner et al., 2013), favouring behavioural flexibility and innovation in short‐distance migrants (Sol et al., 2005). However, while availability of marine and terrestrial resources may be scarce at high latitudes during the winter, some anthropogenic resources (e.g. waste treatment centres) remain dependable year‐round. Such consistency in the environment may limit the need to be plastic, instead favouring reliance on past experience leading to high site fidelity on even fine spatial scales such as we observed. Learned patterns and consistency may be a generally favourable strategy for species utilizing reliable and abundant anthropogenic resources.

## 5 CONCLUSIONS

Due to the challenge migrants face of determining when and where to move without comprehensive knowledge of environmental conditions at remote destinations, concern has been raised regarding whether migrants, particularly long‐distance migrating species who are thought to be more fixed in their behaviour, can sufficiently adjust migratory behaviour to human‐induced environmental change (Møller et al., 2008; Saino et al., 2011). Lesser black‐backed gulls demonstrated consistent individual differences in migratory behaviours, suggesting a preference for relying on past conditions to guide movement, and we found no consistent influence of migration distance on intra‐individual variation. Use of consistent strategies, even by individuals migrating short‐distances who presumably have reliable information regarding current conditions, suggests that familiarity with a strategy may be preferential to trying to track optimal conditions. While this may apply to species who use resources that are predictable year‐round, such as anthropogenic resources (Riotte‐Lambert & Matthiopoulos, 2020), in unpredictable systems a consistent strategy may be detrimental (Abrahms et al., 2018). Importantly, despite an apparent preference for consistency, individuals, regardless of their migration distance, can vary behaviour within the confines for their individual strategies, and occasionally even change strategies. We encourage further examination of the influence of migration distance on behavioural plasticity at the individual‐level to determine how universal our findings are, as well as extending this research to systems where behavioural variation can be linked with environmental variables to assess whether observed behavioural variation is equally adaptive across migration distances.

## AUTHORS' CONTRIBUTIONS

J.M.B., J.S.B. and W.B. conceived the idea for this study; C.J.C., L.L., W.M., C.T. and J.S.B. led the tracking projects; J.M.B. analysed the data under consultation with E.v.L.; J.M.B. wrote the manuscript. All authors contributed critically to the drafts and gave final approval for publication.

## Supporting information

Supplementary Material

## Data Availability

Data (http://doi.org/10.5281/zenodo.3565706, Brown et al., 2019) and R scripts (https://doi.org/10.5281/zenodo.4322799) used in this article are archived and openly available on Zenodo.
