# Supplementary material for: Long‐distance migrants vary migratory behaviour as much as short‐distance migrants: An individual‐level comparison from a seabird species with diverse migration strategies
Source: J Anim Ecol. 2021 Feb 9;90(5):1058–70. doi: 10.1111/1365-2656.13431 (PMC8247866; doi:10.1111/1365-2656.13431)
Supplement: Supplementary file 1 — Supplementary Material [file JANE-90-1058-s001.docx]

**Supplementary material for:** “Long-distance migrants vary migratory behaviour as much as short-distance migrants: an individual-level comparison from a seabird species with diverse migration strategies

Authors: J Morgan Brown, Emiel van Loon, Willem Bouten, Kees CJ Camphuysen, Luc Lens, Wendt Müller, Chris B. Thaxter, Judy Shamoun-Baranes

*
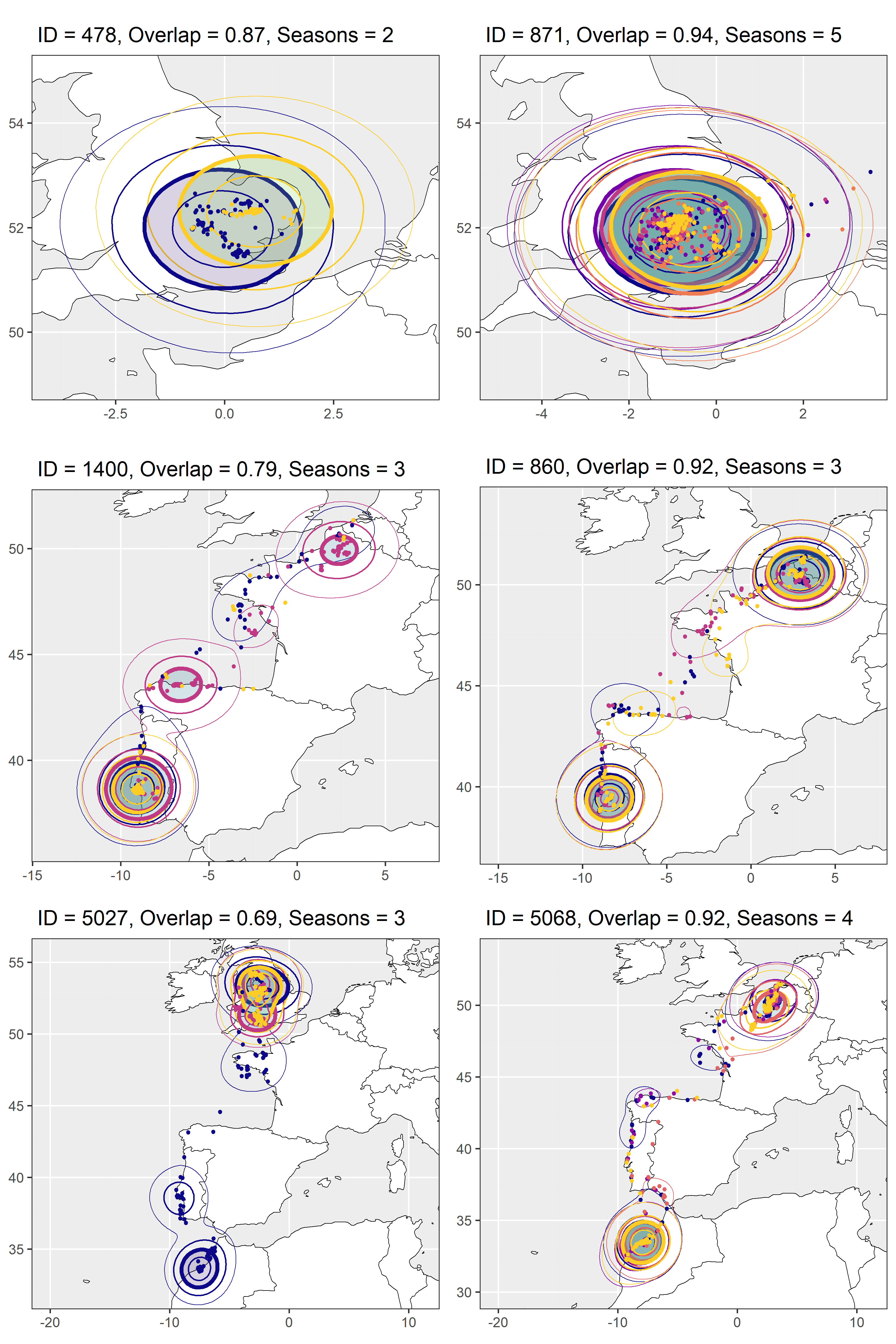
*

**Figure S1.** Examples of relatively low (left) and high (right) nonbreeding distribution overlap for short (top), mid (middle) and long distance migrants (bottom). The 25, 50, 75 and 95% contours are delineated. The 50% contour used to identify core areas are shaded and outlined by the thicker line. The twice daily subsampled points used to make the utilization distributions are shown. Contours and points are coloured by season. The device number (id) and overlap are reported above each plot.

*Methodology: Calculation of mean routes*

Original (non-subsampled) GPS tracks were separated into autumn (from colony departure to arrival at the wintering area) and spring routes (from departure at the wintering area to colony arrival). GPS points between the entrance and exit from a core area (stopovers) were replaced with a single point at the polygon centroid (Fig S1a), smoothing the route. Any migration route with either a gap longer than 24 hours when an individual was outside of a core area (i.e. during migratory flights) or fewer than 10 GPS points remaining on the route were removed. Individuals that did not overlap in their wintering area (n = 5) were excluded from this analysis due to inability to compute a mean route. This resulted in a sample size of 58 individuals in autumn and 69 in spring.

Five-hundred points were created along each GPS route at equally spaced distances (Fig. S1b). To begin computing the mean route, point *i* on the mean route was placed at the mean latitude and longitude of the *i^th^* points from the actual migration routes, resulting in 500 generated points (Fig. S1b). Each point along the mean route was then moved to the mean location of the nearest-neighbour points within ± 1° of latitude on the GPS routes (Fig. S1c). The latitude range was created because, for c-shaped routes, such as around the Iberian peninsula or Bay of Biscay, the nearest neighbour points for the mean route converge on the two points at the mouth of the sea, instead of capturing the shape of the route. Depending on the shape of the migratory routes, mean points may still converge to the same location. If points along the mean route became separated by more than 25 km, a new point was created midway between those two points (Fig S1d). Position averaging and adding of points continued for a total of 100 iterations, resulting in a mean route minimizing the mean squared distance between the GPS tracks (Fig. S1d).

Before calculating variance, 500 equally spaced points were placed along the mean route (total number of points and distances between them become uneven during the computing process). Variance was calculated for each point along the mean route using the distances between the points on the mean route and the nearest-neighbour points on the GPS route (Fig S1e).

*
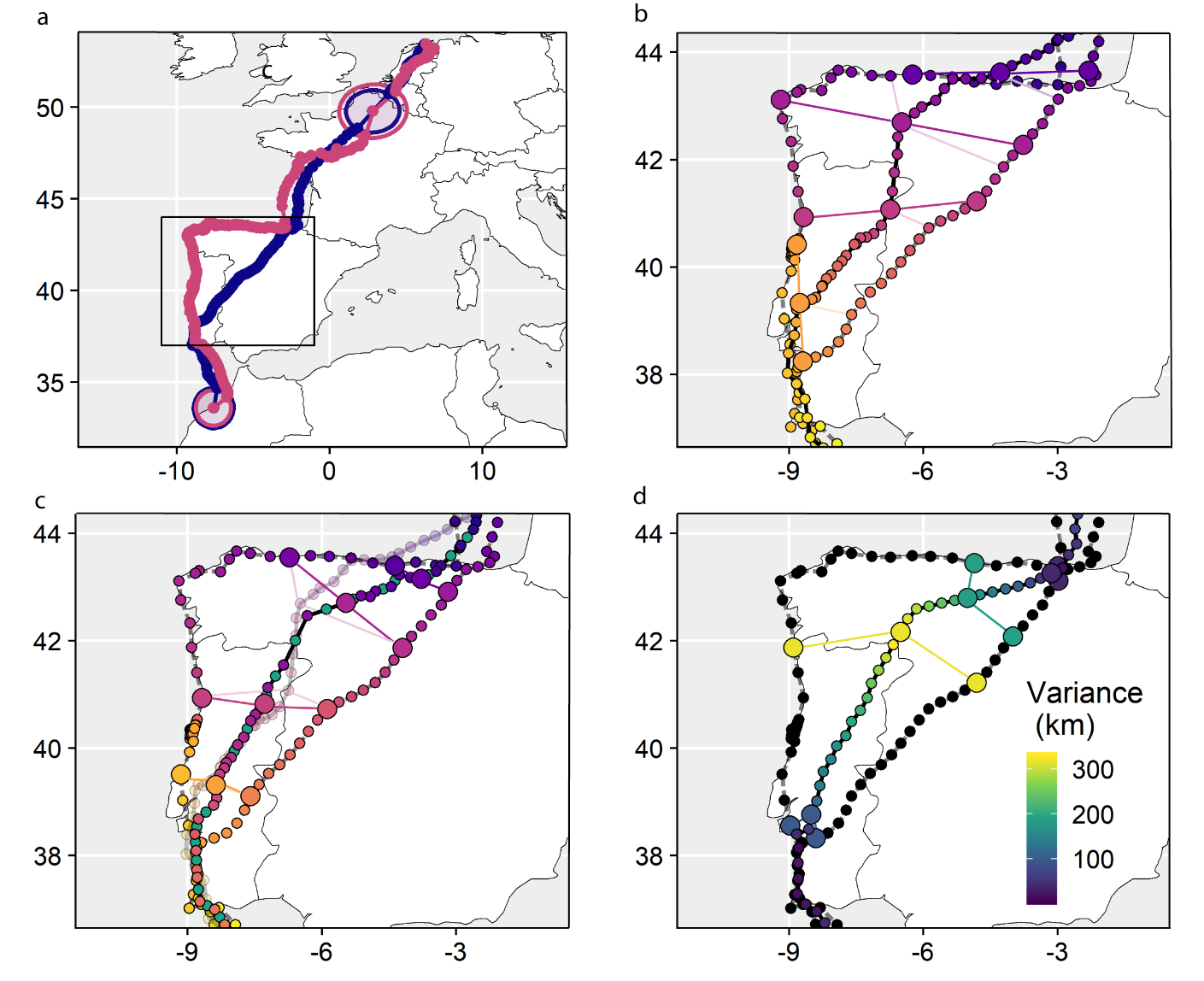
***Figure S2.** An example of how mean routes were computed. Here two deviating routes were used for clarity, though this procedure can be applied to any number of routes. a) The GPS tracks (small points along a line) and core areas (shaded polygons) from ID 5524 during autumn. GPS points within the core area polygons were removed to create a smoothed route. Colour represents different years. Rectangle indicates area shown in b-d. b) A zoomed in section of the routes along which 100 equally spaced points were placed (500 points were used in actual analysis), and the beginning positions of the mean route (centre line). Points are coloured sequentially, such that the colour of the points along the GPS route that were averaged together correspond. Large points connected by lines indicate which points along the GPS tracks were averaged to create the mean route. Faded lines connecting large points on the mean track indicate the nearest-neighbour locations on the GPS track to these points. c) Location of mean route following 1 iteration, with solid line connecting the GPS points averaged to determine the position of select points along the mean route. The original mean route with nearest-neighbour points are indicated by faded points and lines. New points (blueish-green) were added if points along the mean route were separated by more than 25 km. d) The final mean route, after 100 points were equally placed along the route (500 points used in actual analysis). Points on the mean route are coloured based on their variance (low = purple, high = yellow). Lines indicate the distances between mean route and GPS routes used to calculate the variance for select points.

**Table S1.** The number of lesser black-backed gulls with a given number of nonbreeding seasons included in this study (post-processing). As the data processing steps differed depending on the behaviours, the number of individuals and seasons included differed between metrics based on the utilization distributions (and hence the wintering areas), thus migration distance, nonbreeding distribution overlap, and dates of departure and arrival (no gap longer than 21 days), and migration routes (no gap longer than 24 hours during migratory flights). On average 95% of days during a nonbreeding season had a GPS fix (range: 55-100%), and a total of 129 nonbreeding seasons had at least 1 GPS fix per day.

| **Number of Seasons** | **Number of Individuals** | | |
| --- | --- | --- | --- |
|  | **Overlap and timing** | **Autumn route** | **Spring Route** |
| 2 | 39 | 30 | 36 |
| 3 | 25 | 13 | 19 |
| 4 | 13 | 8 | 11 |
| 5 | 3 | 3 | 2 |
| 6 | 1 | 0 | 0 |
| 7 | 1 | 0 | 1 |
| **Total** | **82** | **54** | **69** |

*
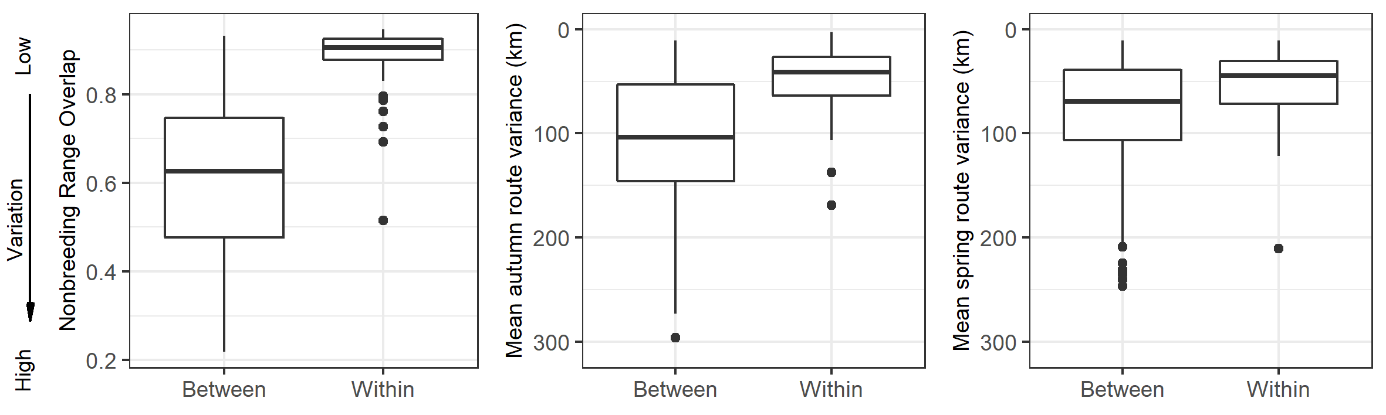
*

**Figure S3**. A comparison of a) nonbreeding season distribution overlap, b) autumn route variation and c) spring route variation within versus between individuals. Differences between the categories (variation ‘between’ and ‘within’ individuals) were highly significant in all three cases, with p < 0.001 based on randomisation tests. The y-axis for autumn and spring route variation (b and c) is reversed so that the direction of variation is consistent with Fig. S3a and Fig 2 in the main manuscript.

*
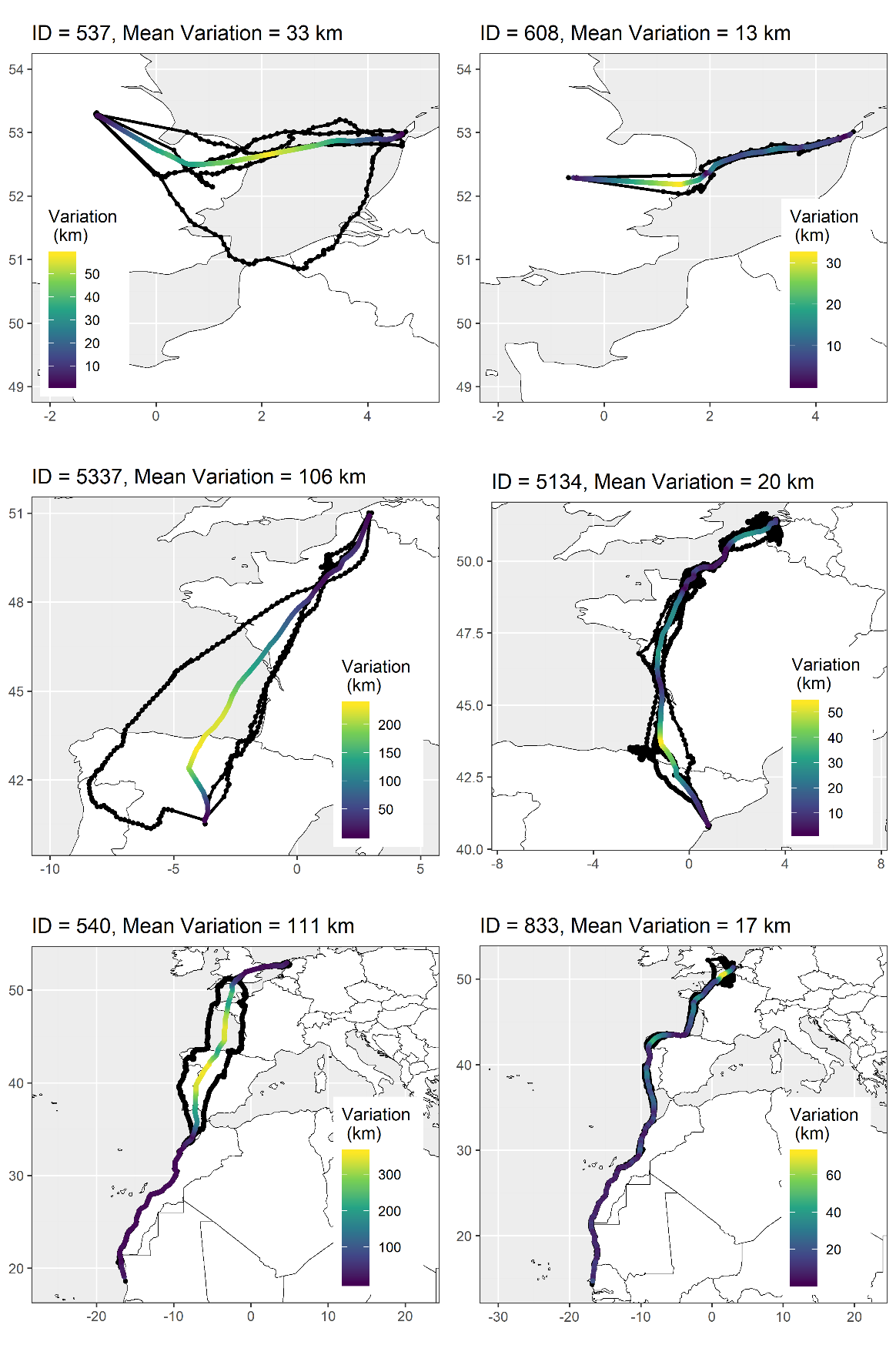
*

**Figure S4.** Examples of relatively high (left) and low (right) route variation for short (top), mid (middle) and long distance migrants (bottom). Black paths show GPS migration routes and mean route is coloured based on variation (scale differs per plot). The device number (id) and mean variation are reported above each plot.

**Table S2.** Linear mixed model results for arrival and departure dates against migration distance with individual and colony as random effects. T-values are reported in brackets for fixed effects. Colony variance is the variance of the colony-level random effect, and individual variance is the variance of the individual-level random effect. Number of observations, n, and number of individuals, n_id_ used in the models are reported under each behaviour. Numbers differ because some individuals were removed due to gaps coinciding with departure or arrival time.

| **Behaviour** | **Intercept ± SE** | **Migration Distance (1000 km) ± SE** | **Colony variance** | **Individual variance** | **Residual variance** |
| --- | --- | --- | --- | --- | --- |
| Colony Departure  n = 227,  n_id_ = 79 | 210.77 ± 5.51 (38.25) | 1.73 ± 2.27 (0.76) | 43.12 | 410.65 | 357.63 |
| Winter Arrival  n = 225,  n_id_ = 78 | 291.72 ± 6.47 (45.10) | - | 44.88 | 2430.72 | 698.14 |
| Winter Departure  n = 229,  n_id_ = 80 | 62.74 ± 3.41 (18.40) | 4.55 ± 1.16 (3.91) | 35.84 | 134.06 | 61.55 |
| Colony Arrival  n = 229,  n_id_ = 80 | 63.16 ± 3.21 (19.67) | 11.10 ± 1.04 (10.71) | 36.41 | 108.37 | 46.15 |

**
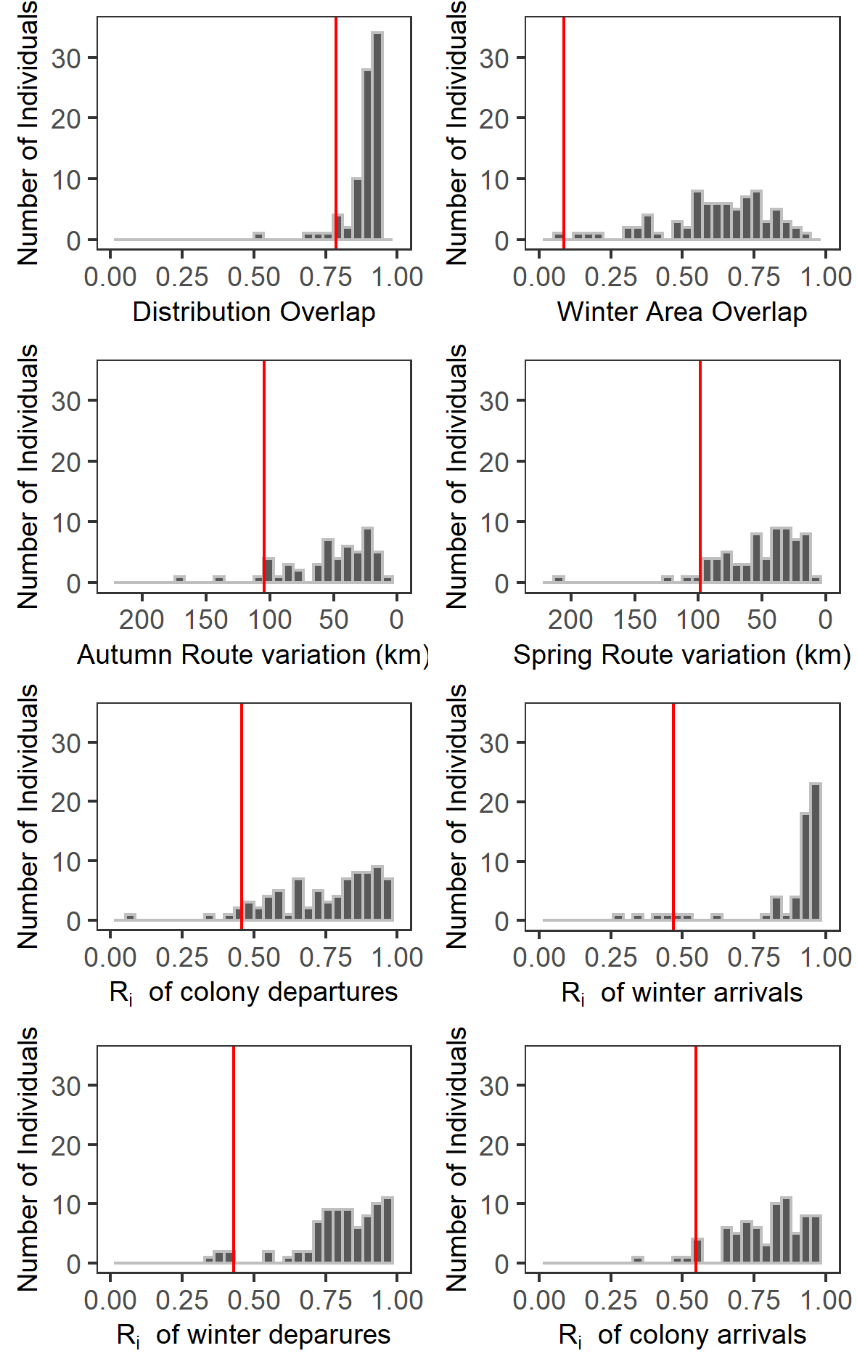
**

c

d

e

g

h

f

a

b

**Figure S5.** Histograms of a) nonbreeding season overlap, b) wintering area site fidelity, c) autumn route variation, d) spring route variation, e) Individual-level repeatability (R_i_) of departure date from colony, f) R_i_ of arrival date to wintering area, g) R_i_ of departure date from wintering area and h) R_i_ of arrival date to colony. Lines indicate the 95^th^ percentile used to identify the most variable individuals in Table S4. Note: x-axis in plots c and d are reversed so that the most variable individuals are on the left, consistent with the other subplots.

**Table S3**. Individuals (device number) in and above the 95^th^ percentile for each measure of variation in the indicated behaviour. Individuals that were among the most variable for multiple behaviours are in bold. Asterisks indicate individuals that did not overlap in their wintering area in one year. All values for overlap and repeatability (R_i_) range from 0 (high variation) to 1 (no variation).

| **Behaviour** | **Individual** | **Individual overlap/variability/repeatability** |
| --- | --- | --- |
| Nonbreeding distribution overlap | **5593*** | 0.51 |
|  | 5027* | 0.69 |
|  | **5555** | 0.73 |
|  | **854** | 0.76 |
|  | **1400** | 0.79 |
| Winter area site fidelity overlap | **606*** | 0.00 |
|  | **1402*** | 0.00 |
|  | **5593*** | 0.00 |
|  | **5555** | 0.00 |
|  | 478 | 0.08 |
| Autumn Route Variability (km) | 5033 | 169 km |
|  | 4024 | 136 km |
|  | 5337 | 106 km |
| Spring Route Variability (km) | 5065 | 211 km |
|  | **5009** | 121 km |
|  | 540 | 112 km |
|  | 5496 | 102 km |
| R_i_ of colony departure date | **854** | 0.05 |
|  | 833 | 0.33 |
|  | 4047 | 0.41 |
|  | 484 | 0.45 |
|  | 853 | 0.46 |
| R_i_ of winter arrival date | **1402*** | 0.265 |
|  | **606*** | 0.349 |
|  | **5027*** | 0.422 |
|  | 534* | 0.44 |
| R_i_ of winter departure date | **606*** | 0.337 |
|  | **1402*** | 0.381 |
|  | **5009** | 0.37 |
|  | 5334 | 0.40 |
|  | **5071** | 0.41 |
| R_i_ of colony arrival date | 503 | 0.35 |
|  | 608 | 0.51 |
|  | **1400** | 0.53 |
|  | 5213 | 0.55 |
|  | 5550 | 0.546 |


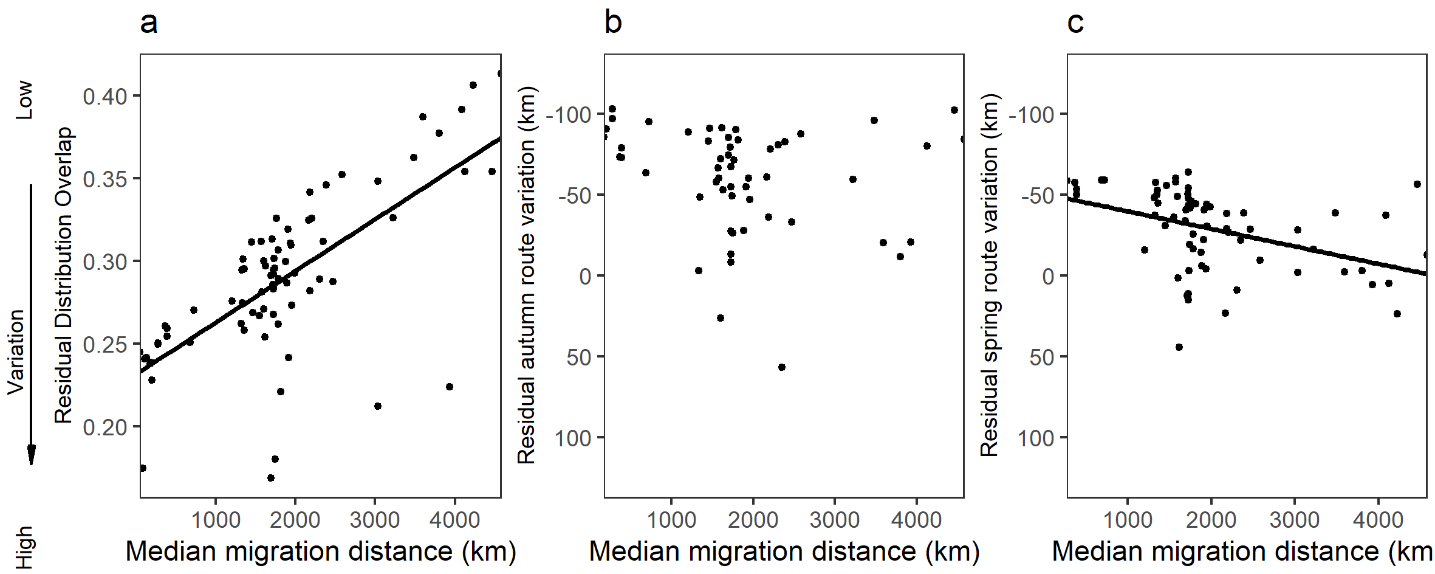


**Figure S6.** a) Residual non-breeding distribution overlap, and residual migration route variation in b) autumn and c) spring across multiple non-breeding seasons from lesser black-backed gulls, versus their mean migration distance. Please note: the y-axis for autumn and spring route variation (b and c) is reversed so that the order of variation is consistent (less variation at the top of the plot). Black line showing trend predicted by the linear models were included if significant. Negative residuals in figures b and c mean that within-individual variation is lower than between individual variation. Individuals who changed wintering areas (n = 5) have been excluded.
